# Crystal structure of 4,4′-bipyridine-1,1’-diium naphthalene-2,6-di­sulfonate dihydrate

**DOI:** 10.1107/S160053681401784X

**Published:** 2014-08-09

**Authors:** Sabri Çevik, Musa Sarı, Murat Sarı, Tuncay Tunç

**Affiliations:** a Department of Chemistry, Faculty of Sciences, Afyon Kocatepe University, 03200 Afyonkarahisar, Turkey; b Gazi University, Department of Physics Education, Beşevler 06500 Ankara, Turkey; c Aksaray University, Faculty of Education, 68100 Aksaray, Turkey

**Keywords:** crystal structure, mol­ecular salt, bi­pyridine, nathphalenedi­sulfonate, dihydrate

## Abstract

The title hydrated mol­ecular organic salt, C_10_H_10_N_2_^2+^·C_10_H_6_O_6_S_2_^2−^·2H_2_O, crystallized with half a bipyridinium cation, half a naphthalene-2,6-di­sulfonate anion and a water mol­ecule in the asymmetric unit. The whole cation and anion are generated by inversion symmetry, the inversion centers being at the center of the bridging C—C bond of the cation, and at the center of the fused C—C bond of the naphthalene group of the anion. In the crystal, the anions and cations stack alternately along the *a* axis with π–π inter­actions [inter-centroid distance = 3.491 (1) Å]. The anions are linked *via* O—H⋯O(sulfonate) hydrogen bonds involving two inversion-related water mol­ecules, forming chains along [10-1]. These chains are bridged by bifurcated N—H⋯(O,O) hydrogen bonds, forming a three-dimensional framework structure. There are also C—H⋯O hydrogen bonds present, reinforcing the framework structure.

## Related literature   

For the use of 4,4′-bi­pyridine in the construction of metal-organic frameworks, see: Batten *et al.* (2012 ▶); Burd *et al.* (2012 ▶); Jeazet & Janiak (2012 ▶). For the use of naphthalene-2,6-di­sulfonate in the preparation of metal-organic frameworks, exploiting its different coordination modes, see: Zhao *et al.* (2013 ▶); Borodkin *et al.* (2013 ▶); Chen *et al.* (2001 ▶); Song *et al.* (2010 ▶); Pereira Silva *et al.* (2006 ▶).
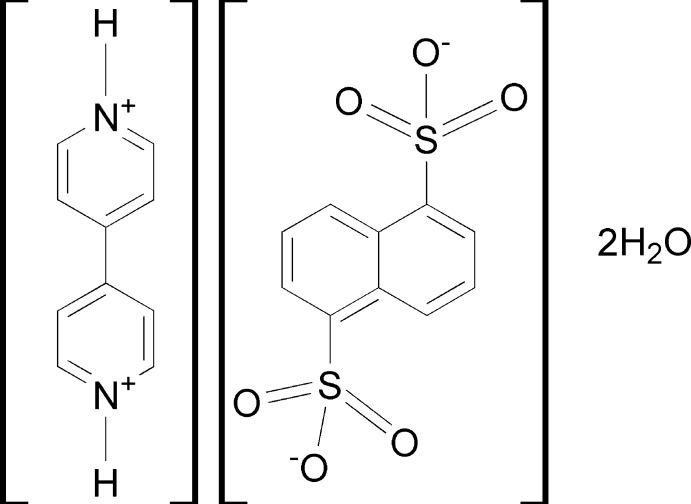


## Experimental   

### Crystal data  

C_10_H_10_N_2_^2+^·C_10_H_6_O_6_S_2_^2−^·2H_2_O
*M* *_r_* = 480.50
Monoclinic, 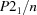

*a* = 7.4022 (2) Å
*b* = 10.9390 (3) Å
*c* = 12.6500 (4) Å
β = 99.908 (1)°
*V* = 1009.03 (5) Å^3^
*Z* = 2
Mo *K*α radiation
μ = 0.32 mm^−1^
*T* = 296 K
0.35 × 0.24 × 0.15 mm

### Data collection  

Bruker SMART BREEZE CCD diffractometer
Absorption correction: multi-scan (*SADABS*; Bruker, 2012 ▶) *T*_min_ = 0.912, *T*_max_ = 0.953
39553 measured reflections
2551 independent reflections
2340 reflections with *I* > 2σ(*I*)
*R*_int_ = 0.033

### Refinement  

*R*[*F* ^2^ > 2σ(*F* ^2^)] = 0.035
*wR*(*F* ^2^) = 0.103
*S* = 1.04
2551 reflections
153 parameters
H atoms treated by a mixture of independent and constrained refinement
Δρ_max_ = 0.41 e Å^−3^
Δρ_min_ = −0.23 e Å^−3^

### 

Data collection: *APEX2* (Bruker, 2012 ▶); cell refinement: *APEX2* and *SAINT* (Bruker, 2012 ▶); data reduction: *SAINT*; program(s) used to solve structure: *SHELXS97* (Sheldrick, 2008 ▶); program(s) used to refine structure: *SHELXL2013* (Sheldrick, 2008 ▶); molecular graphics: *ORTEP-3 for Windows* (Farrugia, 2012 ▶) and *PLATON* (Spek, 2009 ▶); software used to prepare material for publication: *SHELXL2013* and *PLATON* (Spek, 2009 ▶).

## Supplementary Material

Crystal structure: contains datablock(s) global, I. DOI: 10.1107/S160053681401784X/su2764sup1.cif

Structure factors: contains datablock(s) I. DOI: 10.1107/S160053681401784X/su2764Isup2.hkl

Supporting information file. DOI: 10.1107/S160053681401784X/su2764Isup3.cml

**Fig. 1.**
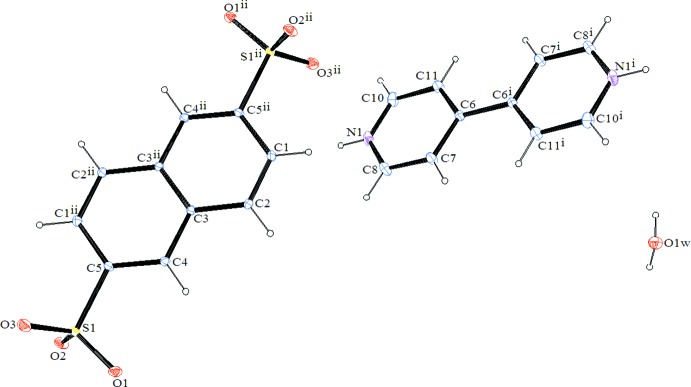
A view of the molecular structure of the title hydrated molecular salt, with the atom labelling. Displacement ellipsoids are drawn at the 40% probability level. [Symmetry codes: (i) -x+1, -y+1, -z+2; (ii) -x+1, -y, -z+1.] The inversion related water molecule is not shown.

**Fig. 2.**
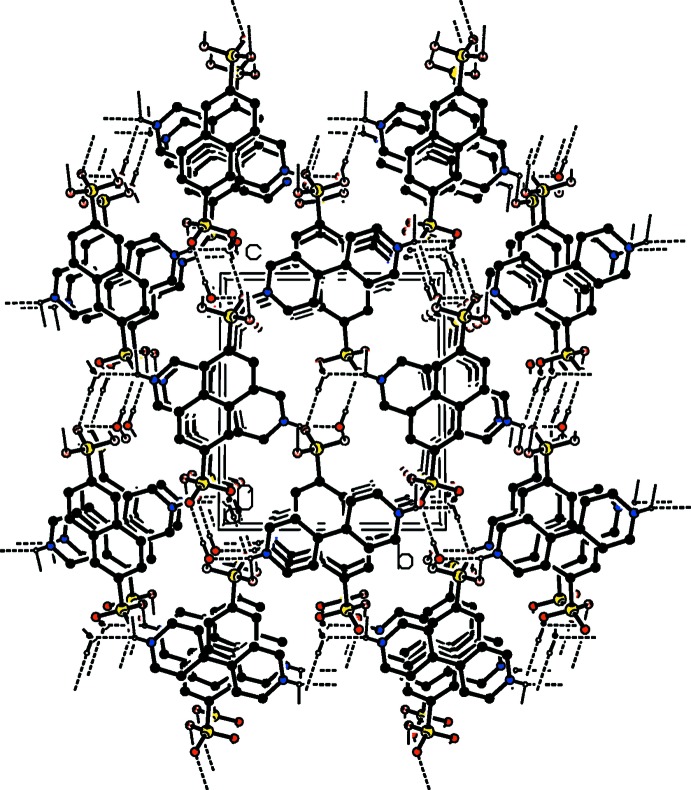
A view along the a axis of the crystal packing of the title compound, showing the hydrogen bonds as dashed lines (see Table 1 for details; H atoms not involved in hydrogen bonds have been omitted for clarity).

. DOI: 10.1107/S160053681401784X/su2764fig1.tif
A view of the mol­ecular structure of the title hydrated mol­ecular salt, with the atom labelling. Displacement ellipsoids are drawn at the 40% probability level. [Symmetry codes: (i) −x+1, −y+1, −z+2; (ii) −x+1, −y, −z+1.] The inversion related water mol­ecule is not shown.

a . DOI: 10.1107/S160053681401784X/su2764fig2.tif
A view along the *a* axis of the crystal packing of the title compound, showing the hydrogen bonds as dashed lines (see Table 1 for details; H atoms not involved in hydrogen bonds have been omitted for clarity).

CCDC reference: 1006095

Additional supporting information:  crystallographic information; 3D view; checkCIF report

## Figures and Tables

**Table 1:** Hydrogen-bond geometry (Å, °)

*D*—H⋯*A*	*D*—H	H⋯*A*	*D*⋯*A*	*D*—H⋯*A*
O1*W*—H1*A*⋯O1^i^	0.82 (3)	2.01 (3)	2.8293 (18)	176 (2)
O1*W*—H1*B*⋯O3^ii^	0.82 (3)	2.04 (3)	2.8484 (18)	172 (2)
N1—H3⋯O2^iii^	0.86	2.55	3.0212 (18)	116
N1—H3⋯O1*W* ^iv^	0.86	1.98	2.7948 (19)	157
C1—H1⋯O1^v^	0.93	2.51	3.1946 (17)	130
C10—H10⋯O1^vi^	0.93	2.60	3.294 (2)	132
C11—H11⋯O2^vii^	0.93	2.47	3.2036 (19)	136

Symmetry codes: (i) 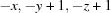; (ii) 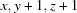; (iii) 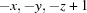; (iv) 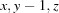; (v) 
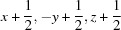
; (vi) 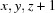; (vii) 
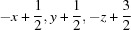
.

## supplementary crystallographic information

### S1. Synthesis and crystallization

The title compound was prepared by a hydro­thermal reaction using a 23 ml Parr Teflon-lined acid digestion bomb, heated in a Nuve brand FN300 model programmable electric oven. A mixture of VOSO_4_·*x*H_2_O, naphthalene-2,6-di­carb­oxy­lic acid, 4,4'-bi­pyridine and water (in molar ratio 1:1:1:55.6 mmol) was placed in a 23 ml Parr Teflon-lined autoclave which was subsequently heated for 90 h inside the oven at 473 K. After, the acid digestion bomb was cooled to room temperature over a period of 4 h. Very-light-yellow–green prismatic single crystals of the title compound and a dark-green unidentified vanadium oxide powder were filtered from the light-blue–green mother liquor. The crystals were separated from the powder under an optical microscope. Analysis calculated for C_20_H_20_N_2_O_8_V_2_: C 49.991, H 4.195, N 5.831, S 13.346%; found: C 48.670, H 3.929, N 5.857, S 13.670%. Selected FT–IR peaks between 1650 and 400 cm^-1^: 1635 (*m*), 1624 (*m*), 1591 (*m*), 1591 (*w*), 1502 (*w*), 1487 (*m*), 1372 (*m*), 1333 (*w*), 1314 (*w*), 1276 (*w*), 1244 (*m*), 1210 (*s*, *sh*), 1200 (*s*), 1194 (*s*, sh), 1183 (*m*, *sh*), 1144 (*m*), 1092 (*s*), 1029 (*s*), 1005 (*m*), 979 (*m*), 914 (*m*), 832 (*m*), 791 (*m*), 706 (*w*), 665 (*s*), 656 (*s*), 618 (*s*), 554 (*m*), 546 (*m*), 507 (*w*), 440 (*m*), 411 (*m*) cm^-1^. Thermogravimetric analysis results supported the X-ray single-crystal structure analysis by giving a 7.5% weight loss in the range 393–413 K, corresponding to two water molecules per molecular formula.

### S2. Refinement

The water molecule H atoms were located in a difference Fourier map and freely refined. The N- and C-bound H atoms were placed geometrically and refined using a riding model, with N—H = 0.86 and C—H = 0.93 Å and with *U*_iso_(H) = 1.2*U*_eq_(N,C).

### Crystal data

**Table:**

C_10_H_10_N_2_^2+^·C_10_H_6_O_6_S_2_^2^^−^·2H_2_O	*F*(000) = 500
*M**_r_* = 480.50	*D*_x_ = 1.581 Mg m^−^^3^
Monoclinic, *P*2_1_/*n*	Mo *K*α radiation, λ = 0.71073 Å
*a* = 7.4022 (2) Å	Cell parameters from 39513 reflections
*b* = 10.9390 (3) Å	θ = 2.5–28.5°
*c* = 12.6500 (4) Å	µ = 0.32 mm^−^^1^
β = 99.908 (1)°	*T* = 296 K
*V* = 1009.03 (5) Å^3^	Plate, colourless
*Z* = 2	0.35 × 0.24 × 0.15 mm

### Data collection

**Table:**

Bruker SMART BREEZE CCD diffractometer	2551 independent reflections
Radiation source: fine-focus sealed X-ray tube	2340 reflections with *I* > 2σ(*I*)
Mirror monochromator	*R*_int_ = 0.033
ω–scans	θ_max_ = 28.5°, θ_min_ = 2.5°
Absorption correction: multi-scan (*SADABS*; Bruker, 2012)	*h* = −9→9
*T*_min_ = 0.912, *T*_max_ = 0.953	*k* = −14→14
39553 measured reflections	*l* = −16→16

### Refinement

**Table:**

Refinement on *F*^2^	Primary atom site location: structure-invariant direct methods
Least-squares matrix: full	Secondary atom site location: difference Fourier map
*R*[*F*^2^ > 2σ(*F*^2^)] = 0.035	Hydrogen site location: mixed
*wR*(*F*^2^) = 0.103	H atoms treated by a mixture of independent and constrained refinement
*S* = 1.04	*w* = 1/[σ^2^(*F*_o_^2^) + (0.0614*P*)^2^ + 0.3867*P*] where *P* = (*F*_o_^2^ + 2*F*_c_^2^)/3
2551 reflections	(Δ/σ)_max_ < 0.001
153 parameters	Δρ_max_ = 0.41 e Å^−^^3^
0 restraints	Δρ_min_ = −0.23 e Å^−^^3^

### Special details

**Table:**

Geometry. All esds (except the esd in the dihedral angle between two l.s. planes) are estimated using the full covariance matrix. The cell esds are taken into account individually in the estimation of esds in distances, angles and torsion angles; correlations between esds in cell parameters are only used when they are defined by crystal symmetry. An approximate (isotropic) treatment of cell esds is used for estimating esds involving l.s. planes.

### Fractional atomic coordinates and isotropic or equivalent isotropic displacement parameters (Å^2^)

**Table:**

	*x*	*y*	*z*	*U*_iso_*/*U*_eq_	
S1	0.21510 (4)	−0.06706 (3)	0.18311 (2)	0.02743 (12)	
O1	0.14850 (17)	0.05494 (9)	0.15242 (9)	0.0398 (3)	
O2	0.06807 (15)	−0.14963 (10)	0.19836 (9)	0.0421 (3)	
O3	0.33036 (17)	−0.11776 (14)	0.11235 (9)	0.0513 (3)	
C1	0.55029 (19)	0.16047 (12)	0.64530 (11)	0.0305 (3)	
H1	0.5516	0.2306	0.6870	0.037*	
C2	0.45657 (18)	0.15973 (11)	0.54200 (10)	0.0291 (3)	
H2	0.3943	0.2295	0.5138	0.035*	
C3	0.45389 (16)	0.05348 (10)	0.47790 (10)	0.0230 (2)	
C4	0.35783 (17)	0.05061 (11)	0.37022 (10)	0.0254 (2)	
H4	0.2972	0.1200	0.3400	0.030*	
C5	0.35503 (17)	−0.05470 (11)	0.31137 (10)	0.0254 (3)	
N1	0.30960 (19)	0.21624 (13)	0.91922 (12)	0.0454 (3)	
H3	0.2627	0.1463	0.8994	0.054*	
C6	0.46027 (17)	0.43924 (12)	0.98236 (11)	0.0282 (3)	
C7	0.3717 (2)	0.41942 (15)	0.87696 (13)	0.0412 (3)	
H7	0.3633	0.4821	0.8267	0.049*	
C8	0.2972 (2)	0.30668 (18)	0.84798 (14)	0.0493 (4)	
H8	0.2375	0.2935	0.7780	0.059*	
C10	0.3922 (3)	0.23110 (15)	1.01926 (15)	0.0479 (4)	
H10	0.3989	0.1662	1.0673	0.057*	
C11	0.4686 (2)	0.34212 (14)	1.05306 (12)	0.0398 (3)	
H11	0.5261	0.3519	1.1239	0.048*	
O1W	0.23786 (19)	0.96590 (12)	0.89658 (11)	0.0447 (3)	
H1A	0.126 (4)	0.958 (2)	0.8846 (19)	0.058 (7)*	
H1B	0.275 (3)	0.940 (2)	0.957 (2)	0.061 (7)*	

### Atomic displacement parameters (Å^2^)

**Table:**

	*U*^11^	*U*^22^	*U*^33^	*U*^12^	*U*^13^	*U*^23^
S1	0.03049 (19)	0.02730 (19)	0.02227 (18)	−0.00197 (11)	−0.00176 (12)	−0.00094 (10)
O1	0.0471 (6)	0.0292 (5)	0.0372 (6)	−0.0022 (4)	−0.0096 (5)	0.0062 (4)
O2	0.0435 (6)	0.0385 (6)	0.0391 (6)	−0.0158 (4)	−0.0076 (4)	0.0034 (4)
O3	0.0470 (7)	0.0765 (9)	0.0290 (5)	0.0134 (6)	0.0025 (5)	−0.0100 (5)
C1	0.0381 (7)	0.0221 (6)	0.0293 (6)	0.0007 (5)	0.0005 (5)	−0.0037 (5)
C2	0.0352 (6)	0.0200 (5)	0.0301 (6)	0.0036 (5)	−0.0003 (5)	0.0002 (5)
C3	0.0241 (5)	0.0198 (5)	0.0246 (6)	−0.0009 (4)	0.0024 (4)	0.0014 (4)
C4	0.0269 (6)	0.0217 (5)	0.0259 (6)	−0.0003 (4)	0.0002 (4)	0.0024 (4)
C5	0.0270 (6)	0.0258 (6)	0.0219 (6)	−0.0028 (4)	0.0001 (4)	0.0006 (4)
N1	0.0439 (7)	0.0390 (7)	0.0573 (8)	−0.0149 (6)	0.0200 (6)	−0.0172 (6)
C6	0.0250 (6)	0.0320 (7)	0.0291 (6)	−0.0030 (5)	0.0085 (5)	−0.0050 (5)
C7	0.0474 (9)	0.0421 (8)	0.0324 (7)	−0.0079 (6)	0.0019 (6)	−0.0050 (6)
C8	0.0510 (9)	0.0537 (10)	0.0417 (8)	−0.0144 (8)	0.0037 (7)	−0.0167 (7)
C10	0.0582 (10)	0.0366 (8)	0.0531 (10)	−0.0097 (7)	0.0217 (8)	0.0011 (7)
C11	0.0468 (8)	0.0393 (8)	0.0336 (7)	−0.0079 (6)	0.0081 (6)	0.0003 (6)
O1W	0.0425 (7)	0.0486 (7)	0.0417 (7)	−0.0014 (5)	0.0037 (5)	0.0042 (5)

### Geometric parameters (Å, º)

**Table:**

S1—O3	1.4489 (12)	N1—C10	1.317 (2)
S1—O1	1.4523 (11)	N1—C8	1.331 (3)
S1—O2	1.4525 (11)	N1—H3	0.8595
S1—C5	1.7735 (12)	C6—C11	1.383 (2)
C1—C2	1.3700 (18)	C6—C7	1.398 (2)
C1—C5^i^	1.4136 (17)	C6—C6^ii^	1.491 (2)
C1—H1	0.9300	C7—C8	1.375 (2)
C2—C3	1.4154 (17)	C7—H7	0.9300
C2—H2	0.9300	C8—H8	0.9300
C3—C3^i^	1.420 (2)	C10—C11	1.376 (2)
C3—C4	1.4244 (17)	C10—H10	0.9300
C4—C5	1.3698 (17)	C11—H11	0.9300
C4—H4	0.9300	O1W—H1A	0.82 (3)
C5—C1^i^	1.4135 (17)	O1W—H1B	0.82 (3)
			
O3—S1—O1	113.29 (8)	C1^i^—C5—S1	117.70 (9)
O3—S1—O2	112.23 (8)	C10—N1—C8	121.70 (14)
O1—S1—O2	112.29 (7)	C10—N1—H3	119.2
O3—S1—C5	106.32 (7)	C8—N1—H3	119.1
O1—S1—C5	107.01 (6)	C11—C6—C7	117.33 (13)
O2—S1—C5	105.01 (6)	C11—C6—C6^ii^	121.33 (16)
C2—C1—C5^i^	120.06 (11)	C7—C6—C6^ii^	121.33 (16)
C2—C1—H1	120.0	C8—C7—C6	119.63 (15)
C5^i^—C1—H1	120.0	C8—C7—H7	120.2
C1—C2—C3	120.41 (11)	C6—C7—H7	120.2
C1—C2—H2	119.8	N1—C8—C7	120.56 (15)
C3—C2—H2	119.8	N1—C8—H8	119.7
C2—C3—C3^i^	119.52 (14)	C7—C8—H8	119.7
C2—C3—C4	121.43 (11)	N1—C10—C11	120.35 (16)
C3^i^—C3—C4	119.05 (13)	N1—C10—H10	119.8
C5—C4—C3	119.78 (11)	C11—C10—H10	119.8
C5—C4—H4	120.1	C10—C11—C6	120.44 (15)
C3—C4—H4	120.1	C10—C11—H11	119.8
C4—C5—C1^i^	121.17 (11)	C6—C11—H11	119.8
C4—C5—S1	120.87 (9)	H1A—O1W—H1B	107 (2)
			
C5^i^—C1—C2—C3	−0.1 (2)	O1—S1—C5—C1^i^	−174.50 (11)
C1—C2—C3—C3^i^	1.3 (2)	O2—S1—C5—C1^i^	65.99 (12)
C1—C2—C3—C4	−179.84 (12)	C11—C6—C7—C8	0.2 (2)
C2—C3—C4—C5	−178.61 (12)	C6^ii^—C6—C7—C8	−178.78 (17)
C3^i^—C3—C4—C5	0.3 (2)	C10—N1—C8—C7	0.3 (3)
C3—C4—C5—C1^i^	−1.51 (19)	C6—C7—C8—N1	−0.4 (3)
C3—C4—C5—S1	172.51 (9)	C8—N1—C10—C11	0.1 (3)
O3—S1—C5—C4	132.64 (12)	N1—C10—C11—C6	−0.3 (3)
O1—S1—C5—C4	11.27 (13)	C7—C6—C11—C10	0.1 (2)
O2—S1—C5—C4	−108.24 (12)	C6^ii^—C6—C11—C10	179.13 (16)
O3—S1—C5—C1^i^	−53.13 (13)		

Symmetry codes: (i) −*x*+1, −*y*, −*z*+1; (ii) −*x*+1, −*y*+1, −*z*+2.

### Hydrogen-bond geometry (Å, º)

**Table:**

*D*—H···*A*	*D*—H	H···*A*	*D*···*A*	*D*—H···*A*
O1*W*—H1*A*···O1^iii^	0.82 (3)	2.01 (3)	2.8293 (18)	176 (2)
O1*W*—H1*B*···O3^iv^	0.82 (3)	2.04 (3)	2.8484 (18)	172 (2)
N1—H3···O2^v^	0.86	2.55	3.0212 (18)	116
N1—H3···O1*W*^vi^	0.86	1.98	2.7948 (19)	157
C1—H1···O1^vii^	0.93	2.51	3.1946 (17)	130
C10—H10···O1^viii^	0.93	2.60	3.294 (2)	132
C11—H11···O2^ix^	0.93	2.47	3.2036 (19)	136

Symmetry codes: (iii) −*x*, −*y*+1, −*z*+1; (iv) *x*, *y*+1, *z*+1; (v) −*x*, −*y*, −*z*+1; (vi) *x*, *y*−1, *z*; (vii) *x*+1/2, −*y*+1/2, *z*+1/2; (viii) *x*, *y*, *z*+1; (ix) −*x*+1/2, *y*+1/2, −*z*+3/2.
