# Supplementary figures and images for: Crystal structure of 4,4′-bipyridine-1,1’-diium naphthalene-2,6-di­sulfonate dihydrate
Source: Acta Crystallogr Sect E Struct Rep Online. 2014 Aug 9;70(Pt 9):o989–90. doi: 10.1107/S160053681401784X (PMC4186165; doi:10.1107/S160053681401784X)

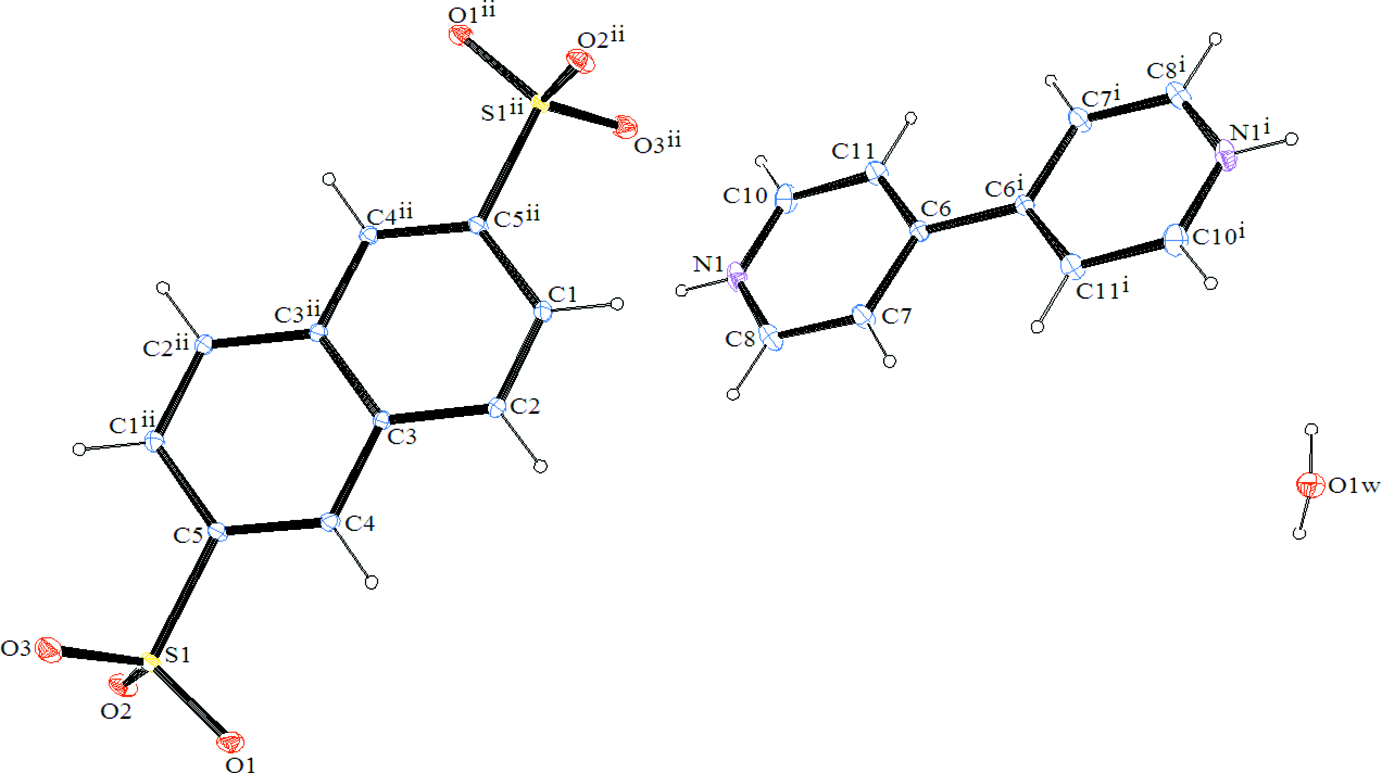

Supplement: Supplementary file 4 [file e-70-0o989-fig1.tif]

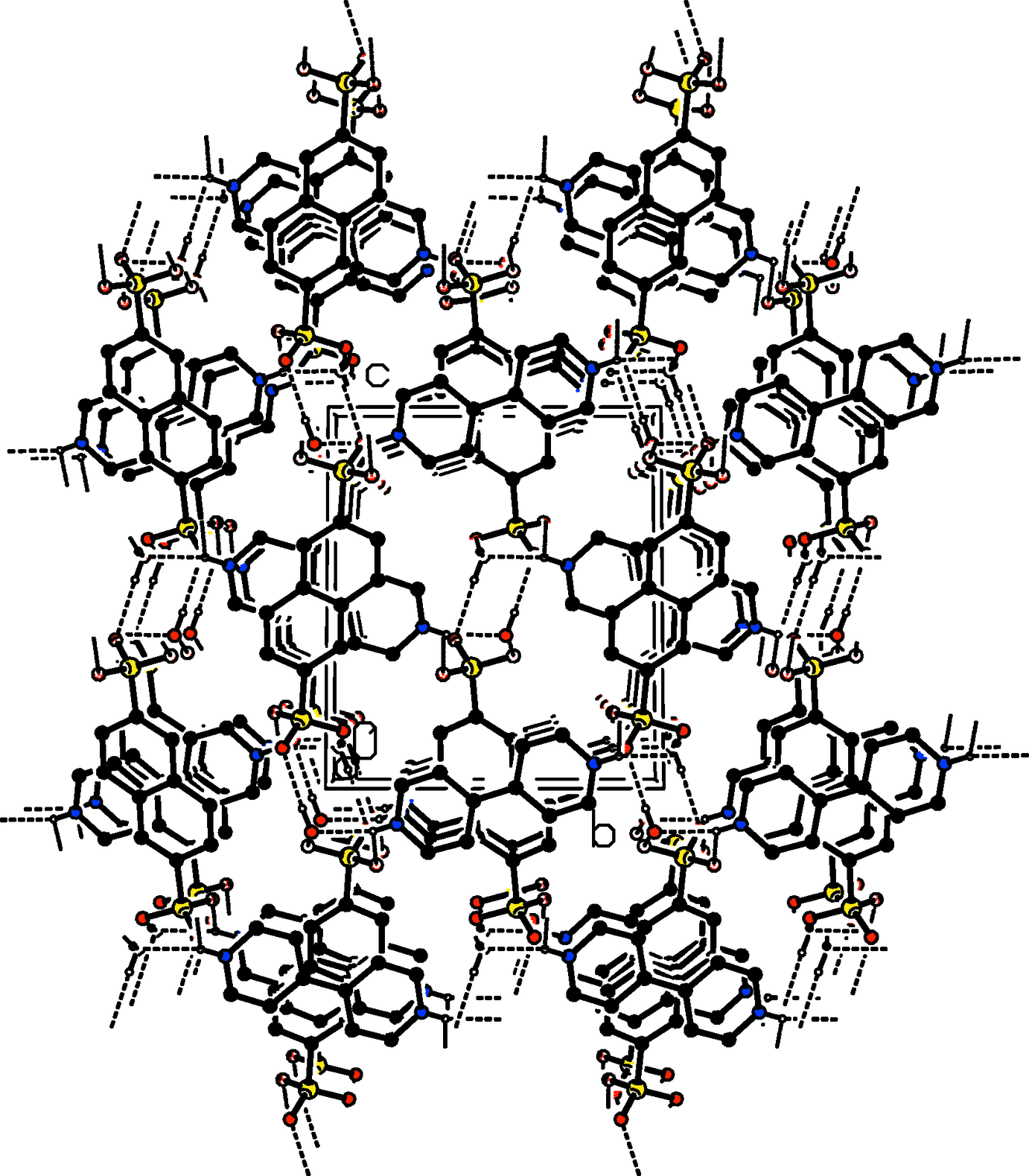

Supplement: Supplementary file 5 [file e-70-0o989-fig2.tif]
